# The evolution of pelvic canal shape and rotational birth in humans

**DOI:** 10.1186/s12915-021-01150-w

**Published:** 2021-10-11

**Authors:** Ekaterina Stansfield, Barbara Fischer, Nicole D. S. Grunstra, Maria Villa Pouca, Philipp Mitteroecker

**Affiliations:** 1 Department of Evolutionary Biology, Unit for Theoretical Biology, University of Vienna, Djerassiplatz 1, 1030 Vienna, Austria; 2 Konrad Lorenz Institute for Evolution and Cognition Research, Martinstrasse 12, 3400 Klosterneuburg, Austria; 3 Mammal Collection, Natural History Museum Vienna, Burgring 7, 1010 Vienna, Austria; 4 Faculty of Engineering of University of Porto (FEUP), Rua Dr. Roberto Frias, s/n, 4200-465 Porto, Portugal; 5 Institute of Science and Innovation in Mechanical and Industrial Engineering (INEGI/LAETA), Rua Dr. Roberto Frias, 400, 4200-465 Porto, Portugal

**Keywords:** Human evolution, Obstetrical dilemma, Pelvis, Rotational birth, Pelvic floor, Biomechanics, Upright posture

## Abstract

**Background:**

The human foetus typically needs to rotate when passing through the tight birth canal because of the complex shape of the pelvis. In most women, the upper part, or inlet, of the birth canal has a round or mediolaterally oval shape, which is considered ideal for parturition, but it is unknown why the lower part of the birth canal has a pronounced anteroposteriorly oval shape.

**Results:**

Here, we show that the shape of the lower birth canal affects the ability of the pelvic floor to resist the pressure exerted by the abdominal organs and the foetus. Based on a series of finite element analyses, we found that the highest deformation, stress, and strain occur in pelvic floors with a circular or mediolaterally oval shape, whereas an anteroposterior elongation increases pelvic floor stability.

**Conclusions:**

This suggests that the anteroposterior oval outlet shape is an evolutionary adaptation for pelvic floor support. For the pelvic inlet, by contrast, it has long been assumed that the mediolateral dimension is constrained by the efficiency of upright locomotion. But we argue that the mediolateral elongation has evolved because of the limits on the anteroposterior diameter imposed by upright posture. We show that an anteroposteriorly deeper inlet would require greater pelvic tilt and lumbar lordosis, which compromises spine health and the stability of upright posture. These different requirements of the pelvic inlet and outlet likely have led to the complex shape of the pelvic canal and to the evolution of rotational birth characteristic of humans.

**Supplementary Information:**

The online version contains supplementary material available at 10.1186/s12915-021-01150-w.

## Background

**Fig. 1:**
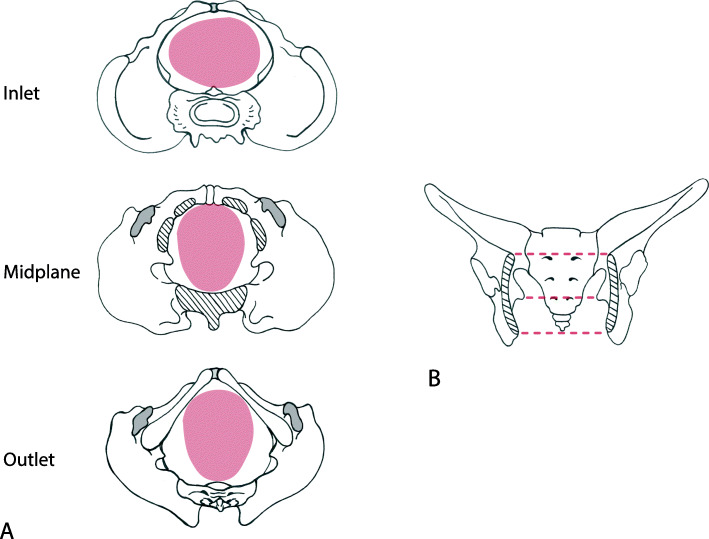
Rotational birth in humans. **A** The foetal head engages in a transverse to oblique direction and rotates about 90° to align its maximum dimension with the largest dimension of each pelvic plane. **B** Pelvic inlet, midplane and outlet in frontal view with parts of the pubic and ischial bones removed

Human childbirth typically involves a complex rotational motion of the foetal head, followed by the shoulders and rest of the body, as the baby passes through the birth canal (Fig. 1A). The tight fit between the human birth canal and the foetus results in relatively high rates of birth-related morbidities and, in the absence of medical interventions, maternal and foetal mortality [1, 2]. Rotational birth is necessary as the human birth canal is not a uniform structure: its largest dimensions are oriented in different directions in the three “planes” of the pelvis, the inlet, the midplane and the outlet (Fig. 1). In most women, the pelvic inlet has its longest diameter in the mediolateral (ML) direction, but the longest diameter in the outlet is in the anteroposterior (AP) direction. In between these planes lies the midplane, which usually is the narrowest part of the human birth canal [3]. This shape difference between the upper and lower birth canal mainly owes to the medially protruding ischial spines in the midplane and the ischial tuberosities as well as the position of the sacrum in the outlet. In physiological vaginal birth, the foetus presents by the head and aligns the largest dimension of the head (the sagittal direction) with the longest diameters of the maternal birth canal in the three planes by rotating through the birth canal (Fig. 1) [4–6]. This raises the question as to why the midplane and the outlet differ in shape from the inlet, thus requiring the complicated and risky rotational birth process. Presumably, human childbirth would be easier if all pelvic planes had the same shape. Great apes, for example, tend to have easier births and their birth canals are both spacious relative to the size of the foetus and have a uniformly anteroposteriorly oval shape [7–10]. Old and New World monkeys also have anteroposteriorly oval-shaped birth canals [8]. Humans are the only primate where the inlet has a mediolaterally oval shape, i.e. an anteroposterior-to-mediolateral ratio (AP/ML) below 1 [8].

Evolutionarily, human pelvic morphology has been the target of many, partly antagonistic selection pressures. The size of the birth canal presumably evolved by trading off the advantage of a large birth canal for childbirth against the disadvantage for bipedal locomotion, thermoregulation, and particularly pelvic floor support [5, 11–20]. The size of the pelvic floor, as determined by the dimensions of the bony pelvis, affects the risk of developing pelvic floor disorders. Up to 45% of women experience some degree of incontinence or pelvic organ prolapse in their life, especially postpartum, but pelvic floor disorders can also affect young and nulliparous women [21–24]. Clinical and biomechanical studies [25–29] confirmed that a larger birth canal increases the risk of pelvic floor disorders, because a larger pelvic floor must be able to bear higher stresses and strains and shows larger vertical displacement under pressure.

We propose that not only the size but also the shape of the birth canal is subject to functional and evolutionary trade-offs between parturition, pelvic floor stability and locomotion. These selective factors, however, differentially affect various parts of the pelvis. The size and shape of the pelvic inlet is particularly decisive for successful parturition. Locomotion efficiency is assumed to be affected by the distance between the acetabula [17, 30], which are located close to the inlet and might thus impose an indirect selection pressure on the inlet form. A narrow distance between the hip joints has been suggested to be energetically more efficient for bipedal locomotion [17, 30], but see [31, 32]. Selection for pelvic floor function acts on the lower birth canal (midplane and outlet), which provides the attachment points for the pelvic floor tissues [11, 33]. A circular or slightly mediolaterally oval inlet (‘gynecoid’ pelvis) is reported to be advantageous for parturition in the gynaecological literature and is the most frequent inlet shape [34–36]. For instance, Betti and Manica [37] reported that the mean AP/ML ratio of the pelvic inlet ranges from 0.77 to 0.94 across 20 human populations, whereas the ratio of AP diameter in the outlet to ML diameter in the midplane (which best represents the dimensions of the pelvic floor) ranges from 1.10 to 1.28. Why is the longest dimension of the lower birth canal not aligned with the longest dimension of the inlet, thus requiring the foetus to perform a complex rotation of the head and shoulders to pass through the birth canal? What is the advantage of a ‘twisted’ birth canal?

The deformation of the pelvic floor in response to pressure increases with the average radius of the pelvic floor for a constant pelvic floor shape [29], which makes women with a larger birth canal more susceptible to pelvic floor dysfunction. When considering variation in pelvic floor shape independent of size, we can idealise the pelvic floor as an elliptically shaped elastic membrane that varies in eccentricity. A circular pelvic floor has a larger minimum diameter than an oval pelvic floor of the same area. In other words, an elliptical shape of the pelvic floor keeps some of the fibres (those along the minor axis of the ellipse) shorter compared to a circular shape of the same area. This, in turn, may reduce pelvic floor deformation under pressure. Based on this argument, we propose that even though a round inlet may be advantageous for childbirth, an oval outlet is advantageous for pelvic floor support.

**Fig. 2:**
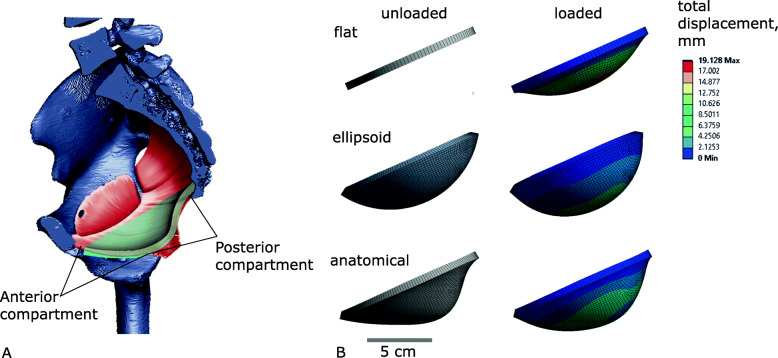
Pelvic floor models (in sagittal view) for finite element analysis. **A** Anatomical model (cyan colour, superimposed on the muscles of the pelvic floor, which are shown in red), oriented approximately along the pubococcygeal axis of an upright person. **B** Flat, ellipsoid and anatomical models (sagittal views). In the left column the three models are shown before loading, whereas the right column shows the displacement in response to 4 kPa pressure. The rainbow colour scheme indicates the magnitude of the maximum displacement of the model elements. Blue corresponds to no displacement, red to high displacement

We tested this hypothesis by a series of finite element analyses (FEA) of idealised pelvic floor models that vary from oval to round, while keeping the area and thickness constant. Loaded with an increased physiological intra-abdominal pressure (typical of a Valsalva manoeuvre, see ‘Methods’ section), we observed the magnitude of deformation (maximum displacement), stress, and strain in the pelvic floor for the differently shaped pelvic floor models. To disentangle the biomechanical effects of pelvic floor geometry from those of the material properties, we studied three different geometric idealisations of the pelvic floor: a flat membrane, a regular 3D oval-shaped hammock, and an anatomically more realistic shape (referred to as ‘flat’, ‘ellipsoid’, ‘anatomical’ models; Fig. 2, see the ‘Methods’ section).

## Results

**Fig. 3:**
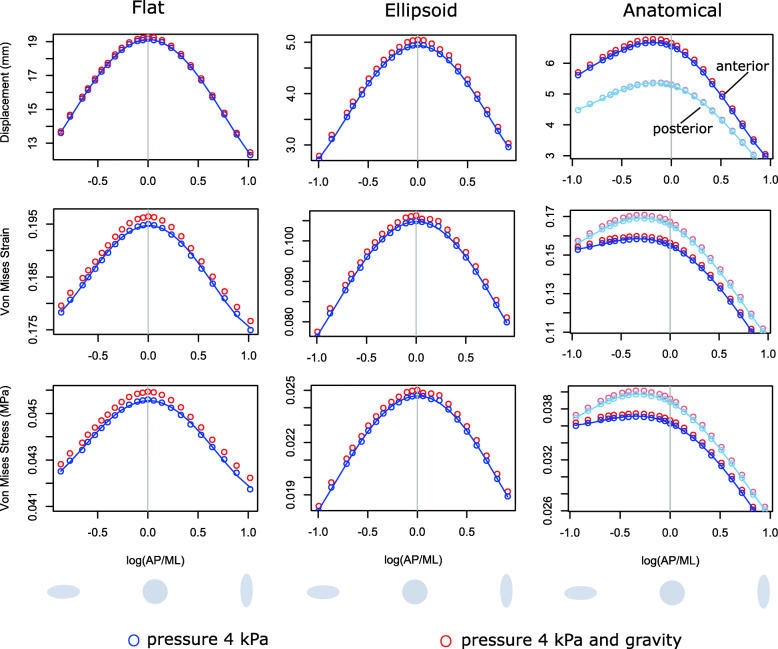
Results of loading experiments (total displacement, maximum von Mises strain, maximum von Mises stress) for the three geometries. In each panel, the horizontal axis represents the shape of the pelvic floor models, expressed as the natural logarithm (log) of the ratio AP/ML to guarantee that the same mediolaterally and anteroposteriorly oval shapes are equally distant from circularity, i.e. log(AP/ML)=-log(ML/AP). Negative values correspond to mediolaterally elongated shapes; positive values to anteroposteriorly elongated shapes (depicted by the grey ellipses below). The value log(AP/ML)=0 corresponds to a circular pelvic floor with AP=ML. The blue marks represent the results for an applied pressure of 4 kPa, and the red marks the results for pressure and gravity applied together. In the anatomical model, most of the deformation occurred in two separate centres, corresponding to the anterior and posterior pelvic floor compartments, for which the results are shown separately here (blue/red for the anterior compartment; light blue/light red for the posterior compartment)

For the flat and ellipsoid models, the highest values of displacement, stress, and strain were found in models with a circular shape (Fig. 3). Introduction of gravity to the finite element analyses increased displacement, stress and strain by a small amount. We modelled gravity by adding the weight of the pelvic floor, applied as an equivalent vertical force, see ‘Methods’ section. For most shapes of the anatomical model, maximum deformations occurred in two separate centres, which correspond to the anterior compartment (between the pubis and the boundary between the anterior and posterior walls of the vagina) and the posterior compartment (posterior wall of the vagina, rectum and post-rectum area) of the pelvic floor (Fig. 2A). Only in the four models with the longest AP diameter did the position of maximum displacement shift away from these compartments (Additional file 1: Fig. S2 and S3). Overall, displacement was higher in the anterior compartment than in the posterior compartment (Fig. 3, see also Additional file 1: Fig. S2 and S3 for the positions of total maximum displacement), whereas strains and stresses were higher in the posterior compartment. In contrast to the flat and ellipsoid models, the highest displacement of the anatomical model occurred at an AP/ML ratio of 0.83, while models with AP/ML=0.71 experienced the highest stresses and strains (Fig. 3). The introduction of gravity slightly increased displacement, stress and strain.

For all three geometries, the stress-strain relationships followed the same trajectory (Additional file 1: Fig. S1). The material stiffness was linear for the experimental pressure of 4 kPa and slightly nonlinear for a pressure of 20 kPa. Nonetheless, also for the higher pressure, all three model types showed the same stress-strain relationship (Additional file 1: Fig. S1). This indicates that the biomechanical differences between anatomical, flat and ellipsoid geometries are due to their differences in shape, rather than due to the non-linear effect of the numerical model of the material (see ‘Methods’ section).

## Discussion

The focus of this study was to investigate how changes in the shape of the pelvic floor—independent of variation in size and tissue properties—affect its stability and displacement under pressure. Therefore, we kept pelvic floor size, thickness and tissue material properties constant in our models, all of which are important clinical risk factors that dominate empirical studies on pelvic floor disorders. Our findings on the influence of pelvic floor shape on its response to pressure are robust with respect to the choice of size and material. Absolute values of displacement, strain and stress would clearly be altered by changes in size, thickness or material, but their relationship with pelvic floor shape, as reported here, would remain similar. Likewise, we assumed tissue isotropy, i.e. we did not explicitly model the different directions of the muscle fibres. Anisotropy of the muscle fibres can affect the degree of deformation at different locations, but they are unlikely to alter the influence of pelvic floor shape on overall deformation. Nonetheless, future modelling work should include both variations in muscle thickness and direction of the muscle fibres.

In agreement with our hypothesis, we found that the ability to resist pressure is indeed affected by the shape of the female pelvic floor, as delimited by the lower birth canal. For flat and ellipsoid models, a circular shape led to the highest displacements. Deviation from circularity in either the anteroposterior (AP) or mediolateral (ML) direction equally reduced deformation, stress and strain. This symmetrical behaviour results from the geometrical symmetry of the flat and ellipsoid models as well as from the isotropic material properties adopted here. For the more realistic anatomical model, by contrast, the sagittal cross-section was not symmetrically shaped, with the maximum curvature located at the area of the anal sphincter rather than at the mid-point. As a result, the highest deformation was not observed for a circular model but for a mediolaterally elongated shape with AP/ML=0.83. The highest values of strain and stress occurred in models with AP/ML=0.71. An even more extreme ML oval shape only weakly reduced displacement, stress and strain. However, increasing the AP/ML ratio towards a more AP oval shape of the anatomical model led to a rapid decrease in all three measures. As our anatomical model still is an idealisation of the real pelvic floor, the actual pelvic floor shape leading to the greatest deformation may deviate from our estimate but is likely to have an AP/ML ratio smaller than 1 (see the ‘Validation’ section in the ‘Methods’ section).

These findings suggest that a mediolaterally elongated shape of the lower birth canal is particularly disadvantageous for pelvic floor support. The more anteroposteriorly oval the lower birth canal is, the more resistant the pelvic floor is in response to pressure. This is in agreement with clinical literature reporting that a mediolaterally wider lower birth canal predisposes to pelvic floor dysfunction [25, 38–40]. Based on these findings, we suggest that the length and orientation of the ischial spines and the sacrum specifically evolved to decouple the shape of the lower birth canal from that of the upper canal in order to ensure a pelvic floor shape that increases the mechanical stability of the pelvic floor. Although non-human primates and other quadrupeds also display anteroposteriorly elongated outlets, they usually have very small ischial spines and a straight and often shorter sacrum [3, 11].

The size of the human pelvic canal is certainly more important for parturition and pelvic floor support than its shape. For instance, the increase in pelvic floor displacement resulting from 1 SD (standard deviation) increase in pelvic floor *size* (Reported by Stansfield et al. [29]) is about 2.8 times as large as the displacement resulting from 1 SD increase in pelvic floor *shape* (AP/ML). Furthermore, the stability of the pelvic floor does not only depend on its size and shape. Parity, mode of delivery, age, obesity and weakness or injuries of pelvic floor tissue are important risk factors for pelvic floor disorders [27, 41–43]. However, most of these factors are presumably uncorrelated with pelvic canal shape and thus are able to evolve independently. Only age is related to both pelvic floor function and pelvic shape [44–47]. But the age at first birth has a low heritability (in a twin study, Topf et al. [48] reported *h*^2^=0.26), and the age changes of the pelvic canal are subtle; it may thus impose little constraints on pelvic evolution. Similarly, although pelvic shape scales allometrically with body height [49], pelvic floor shape is basically uncorrelated with pelvic floor size (*R*^2^=0.002 within populations and *R*^2^=0.028 between human populations, based on the data by Betti & Manica [37]). In other words, the presence of other, clinically more relevant factors does not rule out that the shape of the lower birth canal has an independent effect on pelvic floor stability. In turn, this implies that pelvic floor stability imposes a selective pressure on the shape of the pelvic canal. Although pelvic form is also influenced by nutrition during childhood and adolescence, age of menarche and maternal age at birth, it has a relatively high heritability [50] and thus is expected to respond to the selection imposed by pelvic floor stability.

Our modelling results explain why the lower birth canal evolved an AP oval shape. But they do not explain why the inlet has evolved a different shape and, thus, why the human birth canal is twisted. After all, a uniformly shaped birth canal would likely ease parturition as it would make the complex mode of human rotational birth obsolete. Some rotation during parturition has also been observed in chimpanzees, baboons and squirrel monkeys, all of which have a uniformly AP oval birth canal. In these species, rotation is much simpler and not enforced by the shape of the birth canal but presumably exists to enable the birth of the shoulders [9, 51]. The similarities across non-human primates in birth canal shape suggest that an AP oval inlet constitutes the ancestral primate condition. Why, then, did a mediolaterally shaped pelvic inlet evolve in the human lineage?

**Fig. 4:**
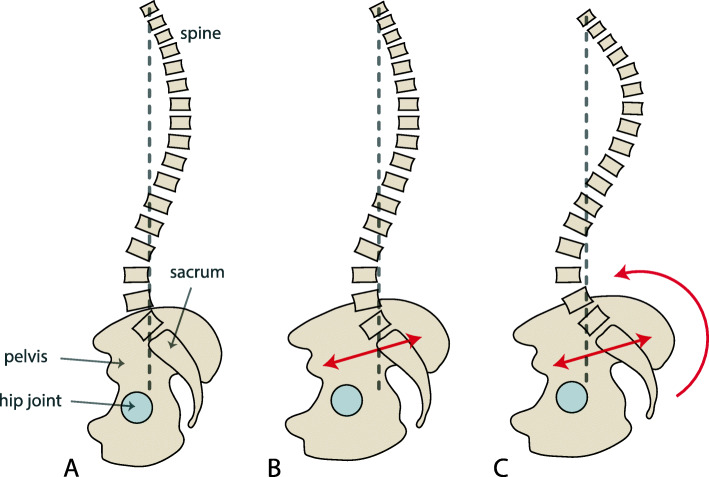
Relationship between pelvic depth (i.e. AP length of the pelvis) and spinal curvature as documented in the orthopaedic literature. The pelvis and spine are shown schematically in sagittal view. **A** Normal spinopelvic relationship where the centre of mass (indicated by the vertical dashed line through the last cervical vertebra, the so-called C7 plumbline) is positioned sagittally above both the hip joints and the superior endplate of the sacrum. **B** In an anteroposteriorly elongated pelvis (as indicated by the red double arrow) without spinal adjustment, the centre of mass is located behind the hip joints, which compromises the structural stability of upright posture. **C** To bring the centre of mass back above the hip joints in this elongated pelvis, the sacrum needs to be tilted forward. This leads to an overall increased curvature of the spine, particularly an increased lumbar lordosis and a deviation of the centre of mass from the sacral endplate. Increased lumbar lordosis is associated with multiple orthopaedic disorders, such as spondylolisthesis and disc herniation

In humans, a balanced upright posture requires a curved spine, particularly a pronounced lumbar lordosis (inward curvature of the lower spine), which brings the centre of mass of the upper body above the line connecting the two hip joints [52, 53]. In this way, the body is pivoted at the hip joints and balanced anteroposteriorly. An increase in AP length of the pelvis would require re-balancing this system by forward-rotating the sacrum and increasing lumbar lordosis (Fig. 4). An increased lumbar lordosis, in turn, may also require increased thoracic and sacral kyphosis (outward curvature). These geometric relationships between spinal curvature and pelvic form are well-documented by the correlations between AP pelvic dimensions, pelvic orientation and lumbar lordosis reported in orthopaedic studies [54–57]. In late pregnancy, lumbar lordosis is even further increased to balance the additional abdominal weight [58]. The amount of spinal curvature, however, is limited by the size, strength and wedging of the vertebral bodies as well as by necessary adaptations within the spinal musculature. It is well known in the orthopaedic clinical literature that a large lordotic angle increases anterior shearing strain in the vertebrae and intervertebral discs and that it brings the centre of mass anterior to the sacral endplate, both of which are associated with chronic back pain, spondylolisthesis (displacement of vertebrae) and disc herniation [59–63].

We therefore suggest that an evolutionary increase in anteroposterior length of the pelvic inlet has been constrained by the adverse effects it would have on spine health and structural stability of upright posture. Since Washburn’s seminal article on the ‘obstetrical dilemma’ [64], researchers have been asking why humans did not evolve a ML wider pelvic inlet to ease birth. Many researchers have assumed that the energetics of efficient upright walking constrain the evolution of a ML wider pelvis [65–67] (although some speculated that also AP dimensions may be constrained by bipedalism [10, 68]). However, the fact that most women *do* have a ML oval inlet implies that the constraint on the ML dimension of the inlet is less severe than that on the AP dimension. Indeed, recent studies found little or no energetic disadvantage associated with a mediolaterally wide pelvis [18] but see [10, 32]. Given this tight biomechanical constraint on the AP diameter of the inlet, a further ML elongation may simply contribute little to ease childbirth. As expected under this hypothesis, the particularly AP narrow pelvis of the bipedal australopithecines [69–72] was likely associated with a lower lordotic angle (41° versus an average of 51° in humans [73]). Chimpanzees, on the contrary, can biomechanically ‘afford’ a pronounced AP oval inlet because they are mostly quadrupedal and do not need to balance their weight above the hip joints.

These spinopelvic relationships also shed light on the human sex differences in lumbar lordosis and vertebral wedging, which tend to be greater in females than in males [58, 74, 75]. Whitcome et al. [58] proposed that this dimorphism, which was already present in early *Homo* and partly even in *Australopithecus*, evolved as an adaptation to mitigate the shearing forces generated by foetal load. However, we suggest that the evolution of increased female lordosis and vertebral wedging are, at least partly, a direct consequence of the larger pelvic canal (including the inlet AP diameter) in females [13, 76]. Only if the average female lordosis exceeds the degree of lordosis expected for female pelvic dimensions would an adaptation for foetal load be a plausible explanation. But this remains to be shown.

Our results provide a novel evolutionary explanation for the twisted shape of the human birth canal. We show that this complex shape has emerged as an evolutionary compromise to different, partly antagonistic selective forces acting on the pelvis: The shape of the birth canal is subject to selection for childbirth, pelvic floor support and upright posture. An even more anteroposteriorly oval lower birth canal would be advantageous for pelvic floor stability but disadvantageous for childbirth. At the same time, an AP oval inlet would ease parturition by avoiding the complex rotation of the foetus but would compromise the structural stability of upright posture and locomotion.

The relative strengths and actual trade-off dynamics of these antagonistic selective forces depend on biological, environmental and sociocultural factors that have changed during human history and partly differ among populations today (‘shifting trade-off model’ [77]). For instance, average pelvic size as well as neonatal weight and head circumference differ considerably across populations, leading to variable magnitudes of obstetric selection on pelvic form [37, 76, 78–80]. The prevalence of pelvic organ prolapse and incontinence vary across countries as well as by ethnicity and sociocultural background [81–83], imposing different strengths of selection for pelvic floor support. Physical activities and diet differ among populations and cultures, thus exerting different physical stresses on the pelvis and the pelvic floor (e.g. [81]) and providing different metabolic capacities during pregnancy [31]. Transitions in environmental and socioeconomic conditions can also affect the relationship between average foetal and maternal size, which influences the difficulty of labour [84, 85]. Hence, it is likely that the observed population differences in pelvic shape [37] have partly resulted from local differences in selective pressures.

## Conclusions

The anteroposterior oval shape of the lower pelvic canal is an evolutionary adaptation for pelvic floor support. By contrast, the mediolateral elongation of the pelvic inlet presumably evolved because of the limits on the anteroposterior diameter imposed by upright posture. We showed that an anteroposteriorly deeper inlet would require greater pelvic tilt and lumbar lordosis, which compromises spine health and the stability of upright posture. These different requirements of the pelvic inlet and outlet likely have led to the complex shape of the pelvic canal and to the evolution of rotational birth characteristic of humans.

## Methods

We assessed how changes in the ratio of anteroposterior to mediolateral diameters (AP/ML) of the pelvic floor affect the amount of displacement under physiological pressure conditions. We used three different idealisations of pelvic floor geometry: a flat membrane, a regular 3D oval-shaped hammock and a 3D membrane that resembles real pelvic floor geometry as it is suspended in the midplane and outlet of the birth canal. We refer to the first model as ‘flat’, the second model as ‘ellipsoid’ and the third model as ‘anatomical’ (Fig. 2). The shape of the transverse outline of each of the three models was varied from mediolaterally wide to anteroposteriorly long and loaded with pressure from above. As the ‘anatomical’ model is geometrically in-between the flat and ellipsoid models, the latter two set the range of the expected mechanical response due to the three-dimensional curvature. The ‘anatomical’ model, with any approximation to the real shape made by us, is therefore expected to experience deformations within this range of behaviour. Due to the symmetry of the flat and ellipsoid models, any deviation of the deformation behaviour in the anatomical pelvic floor will then be possible to ascribe to its more complex 3D geometry.

### 3D geometry

Computer-aided design (CAD) models of the pelvic floor were created as shells in SOLIDWORKS (© 1995-2019 Dassault Systémes). Their mediolateral (ML) diameter corresponded to the distance between the ischial bones at the points of muscle insertion on the ischial spines and was thus equal to the width of the midplane of the birth canal (Fig. 1C). The anteroposterior diameter of each model corresponded to the distance from the inferior point at the pubic symphysis to the apex of the fifth sacral vertebra, which was equal to the length of the outlet of the birth canal in the sagittal view (Fig. 1B).

The transverse diameters of our models were based on the means and standard deviations (SD) of modern Europeans as reported by DelPrete [86]. The ‘base model’ was assigned the average values of the ML and AP diameters. To create further models, we varied the ML diameter in steps of 0.5 or 1.0 standard deviations (SD), while the surface area was kept constant by a corresponding change of the AP diameter. In total, 23 models whose ML diameter ranged from −4.5 to +8 SD from the mean were created for each of the three experiments (i.e. flat, ellipsoid and anatomical; Additional file 1: Tab. S1). This range extends well beyond the variation observable in modern humans, allowing us to assess how extreme pelvic shapes, which may have been selected against in the past, would perform. The ellipsoid and anatomical models were assigned a constant depth of 2.9 mm. The depth was determined as an average value of the perpendicular distance between the pubo-coccygeal axis and the position of the anal sphincter in sagittal CT scans of 10 female individuals chosen randomly from the New Mexico Decedent Image Database collection [87]. The depth of the ellipsoid model was taken perpendicular to the anteroposterior and mediolateral axes at the centre point. The 3D geometry of the anatomical base model was built following the protocol of Stansfield et al. [29]. Figure 1 demonstrates the fit of the three models in the female birth canal. Details of all models are given in the Additional file 1: Table S1.

### Finite element model

#### FE model

We assigned a uniform thickness of 6 mm and a density of 1.0597 g/cm^3^ to each of the models [29, 88]*.* The geometry was discretised using more than 3000 HEX 8 elements with an average element size of 2 mm. An implicit solution scheme using ANSYS Mechanical (© 2008-2021 ANSYS, Inc.) was adopted to solve the quasi-static loading problem. The boundary conditions were identical across models and involved setting the mobility of the rim to zero in all three directions, while allowing for rotation. We assumed that the pelvic floor is at an equilibrium at the normal intra-abdominal pressure of 0.5kPa [89]. The pressure of 4.5kPa is an average intra-abdominal pressure produced by a Valsalva (straining) manoeuvre, a technique used in medical diagnostics, where patients make a forceful exhale motion while keeping airways closed. This increases the intra-abdominal pressure in a controlled way without contraction of the pelvic floor muscles [90]*.* We therefore apply the difference of 4kPa between the normal and the Valsalva intra-abdominal pressure as an equivalent normal force to the entire superior surface of the mesh. This approach allowed us to validate our pelvic floor model (Additional file 1: Tab. S2) against published data on pelvic floor displacement during dynamic magnetic resonance images of healthy women [91]*.* The pressure of 4.5 kPa is evolutionarily relevant as it is within the upper range of normal intra-abdominal pressure during typical activities [89]*.* Across all experiments, material properties were kept constant to assess how displacement, stresses and strains of the pelvic floor changed as a consequence of pelvic floor geometry.

#### Material properties and model validation

We adopted an isotropic Mooney-Rivlin constitutive law (also see Additional file 1) to represent pelvic floor tissues with the following parameters: *c*_*1*_=26 kPa, *c*_*2*_=14 kPa [91], and the bulk modulus, *K =* 1000 kPa [92]. These material properties have been sourced from published literature [91] and were previously used for validation of our anatomical model with the base ML and AP diameters [29] (Additional file 1: Tab. S2).

#### Experiments and measurements

Separately for the flat, ellipsoid and anatomical geometries and the different AP/ML ratios (Additional file 1: Tab. S1), we quantified the mechanical response to the applied pressure of 4kPa. We kept the surface area constant in all experiments. In the ellipsoid and anatomical models, we also kept the depth constant. In addition, we assessed the effect of non-linear material properties by comparing the relationship between stresses and strains in the flat, ellipsoid and anatomical base models when applying experimental pressures of 4 kPa and 20 kPa.

We measured three variables at the location of maximum displacement to assess the performance of the pelvic floor model: the maximum total displacement magnitude (in mm), the maximum von Mises strain and the maximum von Mises stress (in MPa). The engineering term ‘strain’ is a dimensionless measure that describes the amount of extension the material experiences per unit length, while ‘stress’ denotes the amount of force experienced by the material per unit of its surface. A large strain magnitude implies that the material was stretched, compressed or sheared to a high degree. At the same time, a large stress signifies a high amount of energy that the material absorbed in order to achieve its deformation. The von Mises stresses and strains are theoretical values calculated from the three-dimensional stress and strain state of the material, therefore combining in a single parameter the maximum distortion energy [93]. We ignored stresses and strains at the edges of the models because of our boundary conditions. The measurements were exported from ANSYS Mechanical as the maximum value for 20 elements located at and around the centre of the maximum displacement. These elements were identical across results for one model but varied across models due to slight differences in the node numbering and the location of the maximum displacement. In anatomical models, the maximum displacement positions were determined for the anterior and posterior compartments (Additional file 1: Fig. S2). All experiments were performed both with and without the effect of gravity. The latter accounted for the weight of the pelvic floor membrane only and was automatically calculated from the provided density of the material. It was then applied as an equivalent vertical (as opposed to normal) force to the entire volume of the mesh.

#### Validation

The geometry of the real human pelvic floor is more complex than our anatomical model as it is neither homogeneous in thickness nor in tissue properties. Additionally, pelvic floor tissue properties vary due to individual genetic differences and change with age, hormonal status and pelvic floor training [94–100]. They also differ between women with and without urinary incontinence [90–102]. Our base model, however, was successfully validated by measuring the displacement of the posterior compartment against published dynamic magnetic resonance imaging data [29] (Additional file 1: Tab. S2) and captures the essence of the behaviour of the female pelvic floor during a Valsalva manoeuvre by revealing two main areas of displacement. The maximum displacement of the anterior compartment in our model occurs at the anatomical location of the urogenital hiatus, where fibres of the urogenital diaphragm and the anterior part of the levator ani insert into the urethra and anterior vagina. The maximum displacement of the posterior compartment in our model coincides with the location of the ‘bend’ created between the levator plate and the puborectalis muscle (Fig. 2A). In clinical practice, displacements in these two areas are used for diagnosing pelvic floor tissue health and prolapse [103–107], indicating that our base model successfully reproduces the anatomical areas critical for pelvic floor health.

## Supplementary Information

**Additional file 1:** **Tab. S1.** List of models and their AP/ML ratios used for finite element analysis. **Tab. S2.** FE model validation. **Fig. S1.** Stress-strain relationships for the three geometries (i.e., flat, ellipsoid and anatomical) at the base dimensions. **Fig. S2.** Total displacement across all anatomical models. **Fig. S3.** Comparison of displacement maxima at the anterior and posterior compartments of the anatomical models and their total maxima.

## Data Availability

The datasets supporting the conclusions of this article are available in an Open Science Framework (OSF) repository, https://osf.io/9n3db/ [108].

## References

[1] Dolea C, AbouZahr C (2003). Global burden of obstructed labour in the year 2000.

[2] Neilson JP, Lavender T, Quenby S, Wray S (2003). Obstructed labour. Br Med Bull..

[3] Trevathan W (2015). Primate pelvic anatomy and implications for birth. Philos Trans R Soc Lond B Biol Sci..

[4] Rosenberg K, Trevathan W (2002). Birth, obstetrics and human evolution. BJOG..

[5] Wittman AB, Wall LL (2007). The evolutionary origins of obstructed labor: bipedalism, encephalization, and the human obstetric dilemma. Obstet Gynecol Surv..

[6] Collins S, Arulkumaran S, Hayes K, Jackson S. Oxford handbook of obstetrics and gynaecology. Oxford: OUP Oxford; 2008.

[7] Nissen HW, Yerkes RM (1943). Reproduction in the chimpanzee: report on forty-nine births. Anat Rec..

[8] Tague RG (2005). Big-bodied males help us recognize that females have big pelves. Am J Phys Anthropol..

[9] Hirata S, Fuwa K, Sugama K, Kusunoki K, Takeshita H (2011). Mechanism of birth in chimpanzees: humans are not unique among primates. Biol Lett..

[10] Haeusler M, Grunstra NDS, Martin RD, Krenn VA, Fornai C, Webb NM. The obstetrical dilemma hypothesis: there’s life in the old dog yet. Biol Rev Camb Philos Soc. 2021. 10.1111/brv.12744. 10.1111/brv.12744 PMC8518115 34013651

[11] Abitbol M (1988). Evolution of the ischial spine and of the pelvic floor in the Hominoidea. Am J Phys Anthropol..

[12] Lovejoy CO (1988). Evolution of human walking. Sci Am..

[13] Tague RG (1992). Sexual dimorphism in the human bony pelvis, with a consideration of the Neandertal pelvis from Kebara Cave, Israel. Am J Phys Anthropol.

[14] Ruff C (2002). Variation in human body size and shape. Annu Rev Anthropol..

[15] Rosenberg K, Trevathan W (2005). Bipedalism and human birth: the obstetrical dilemma revisited. Evolutionary Anthropol..

[16] Mitteroecker P, Huttegger SM, Fischer B, Pavlicev M (2016). Cliff-edge model of obstetric selection in humans. Proc Natl Acad Sci U S A..

[17] Ruff C (2017). Mechanical constraints on the hominin pelvis and the “obstetrical dilemma.”. Anat Rec..

[18] Gruss LT, Gruss R, Schmitt D (2017). Pelvic breadth and locomotor kinematics in human evolution. Anat Rec.

[19] Grunstra NDS, Zachos FE, Herdina AN, Fischer B, Pavličev M, Mitteroecker P. Humans as inverted bats: a comparative approach to the obstetric conundrum. Am J Hum Biol. 2019;31. 10.1002/ajhb.23227 PMC6492174 30810261

[20] Pavličev M, Romero R, Mitteroecker P (2020). Evolution of the human pelvis and obstructed labor: new explanations of an old obstetrical dilemma. Am J Obstet Gynecol..

[21] Dietz HP, Clarke B (2005). Prevalence of rectocele in young nulliparous women. Aust N Z J Obstet Gynaecol..

[22] Kenton K, Mueller ER (2006). The global burden of female pelvic floor disorders. BJU Int..

[23] Nygaard I, Barber MD, Burgio KL, Kenton K, Meikle S, Schaffer J (2008). Prevalence of symptomatic pelvic floor disorders in US women. JAMA..

[24] Lawrence JM, Lukacz ES, Nager CW, Hsu J-WY, Luber KM (2008). Prevalence and co-occurrence of pelvic floor disorders in community-dwelling women. Obstet Gynecol..

[25] Sze EH, Kohli N, Miklos JR, Roat T, Karram MM (1999). Computed tomography comparison of bony pelvis dimensions between women with and without genital prolapse. Obstet Gynecol..

[26] Handa VL, Pannu HK, Siddique S, Gutman R, VanRooyen J, Cundiff G (2003). Architectural differences in the bony pelvis of women with and without pelvic floor disorders. Obstet Gynecology..

[27] Brown KM, Handa VL, Macura KJ, DeLeon VB (2013). Three-dimensional shape differences in the bony pelvis of women with pelvic floor disorders. Int Urogynecol J..

[28] Sammarco AG, Sheyn D, Hong CX, Kobernik EK, Swenson CW, Delancey JO (2021). Pelvic cross-sectional area at the level of the levator ani and prolapse. Int Urogynecol J..

[29] Stansfield E, Kumar K, Mitteroecker P, Grunstra NDS. Biomechanical trade-offs in the pelvic floor constrain the evolution of the human birth canal. PNAS. 2021;118. 10.1073/pnas.2022159118. 10.1073/pnas.2022159118 PMC8072325 33853947

[30] Lovejoy CO (2005). The natural history of human gait and posture. Gait Posture..

[31] Dunsworth HM, Warrener AG, Deacon T, Ellison PT, Pontzer H (2012). Metabolic hypothesis for human altriciality. Proc Natl Acad Sci U S A..

[32] Warrener AG, Lewton KL, Pontzer H, Lieberman DE (2015). A wider pelvis does not increase locomotor cost in humans, with implications for the evolution of childbirth. PLoS One..

[33] Herschorn S (2004). Female pelvic floor anatomy: the pelvic floor, supporting structures, and pelvic organs. Rev Urol..

[34] Caldwell WE, Moloy HC (1938). Anatomical variation in the female pelvis: their classification and obstetrical significance. Proc R Soc Med.

[35] Maharaj D (2010). Assessing cephalopelvic disproportion: back to the basics. Obstet Gynecol Surv..

[36] Stewart KS, Cowan DB, Philpott RH (1979). Pelvic dimensions and the outcome of trial labour in Shona and Zulu primigravidas. S Afr Med J..

[37] Betti L, Manica A. Human variation in the shape of the birth canal is significant and geographically structured. Proc Biol Sci. 2018;285. 10.1098/rspb.2018.1807. 10.1098/rspb.2018.1807 PMC6234894 30355714

[38] Stav K, Alcalay M, Peleg S, Lindner A, Gayer G, Hershkovitz I (2007). Pelvis architecture and urinary incontinence in women. Eur Urol..

[39] Xu H-N, Xia Z-J, Li B-X, Yin Y-T, Wang F, Hu Q (2011). Investigation of correlation between diameters of pelvic inlet and outlet planes and female pelvic floor dysfunction. Eur J Obstet Gynecol Reprod Biol..

[40] Berger MB, Doumouchtsis SK, DeLancey JO (2013). Bony pelvis dimensions in women with and without stress urinary incontinence. Neurourol Urodyn..

[41] Blomquist JL, Muñoz A, Carroll M, Handa VL (2018). Association of Delivery Mode With Pelvic Floor Disorders After Childbirth. JAMA..

[42] Lukacz ES, Lawrence JM, Contreras R, Nager CW, Luber KM (2006). Parity, mode of delivery, and pelvic floor disorders. Obstet Gynecol..

[43] Wu JM, Vaughan CP, Goode PS, Redden DT, Burgio KL, Richter HE (2014). Prevalence and trends of symptomatic pelvic floor disorders in U.S. women. Obstet Gynecol..

[44] Huseynov A, Zollikofer CPE, Coudyzer W, Gascho D, Kellenberger C, Hinzpeter R (2016). Developmental evidence for obstetric adaptation of the human female pelvis. Proc Natl Acad Sci U S A..

[45] Kolesova O, Kolesovs A, Vetra J (2017). Age-related trends of lesser pelvic architecture in females and males: a computed tomography pelvimetry study. Anat Cell Biol..

[46] Auerbach BM, King KA, Campbell RM, Campbell ML, Sylvester AD (2018). Variation in obstetric dimensions of the human bony pelvis in relation to age-at-death and latitude. Am J Phys Anthropol..

[47] Waltenberger L, Rebay-Salisbury K, Mitteroecker P. Age dependent changes in pelvic shape during adulthood. Homo. (accepted). 10.1127/anthranz/2021/1463 34664055

[48] Tropf FC, Barban N, Mills MC, Snieder H, Mandemakers JJ (2015). Genetic influence on age at first birth of female twins born in the UK, 1919–68. Popul Stud.

[49] Fischer B, Mitteroecker P (2017). Allometry and sexual dimorphism in the human pelvis. Anat Rec.

[50] Sharma K (2002). Genetic basis of human female pelvic morphology: a twin study. Am J Phys Anthropol..

[51] Stoller MK (1996). The obstetric pelvis and mechanism of labor in nonhuman primates.

[52] Latimer B, Ward CV, Walker A, Leakey REF (1993). The thoracic and lumbar vertebrae. The Nariokotome Homo erectus Skeleton.

[53] Shapiro LJ (1993). Functional morphology of the vertebral column in primates.

[54] Roussouly P, Pinheiro-Franco JL (2011). Biomechanical analysis of the spino-pelvic organization and adaptation in pathology. Eur Spine J..

[55] Boulay C, Bollini G, Legaye J, Tardieu C, Prat-Pradal D, Chabrol B (2014). Pelvic incidence: a predictive factor for three-dimensional acetabular orientation-a preliminary study. Anat Res Int..

[56] Laouissat F, Sebaaly A, Gehrchen M, Roussouly P (2018). Classification of normal sagittal spine alignment: refounding the Roussouly classification. Eur Spine J..

[57] Pizones J, García-Rey E (2020). Pelvic motion the key to understanding spine-hip interaction. EFORT Open Rev..

[58] Whitcome KK, Shapiro LJ, Lieberman DE (2007). Fetal load and the evolution of lumbar lordosis in bipedal hominins. Nature..

[59] von Lackum HL (1924). The lumbosacral region: an anatomic study and some clinical observations. JAMA..

[60] Labelle H, Mac-Thiong J-M, Roussouly P (2011). Spino-pelvic sagittal balance of spondylolisthesis: a review and classification. Eur Spine J..

[61] Caglayan M, Tacar O, Demirant A, Oktayoglu P, Karakoc M, Cetin A (2014). Effects of lumbosacral angles on development of low back pain. J Musculoskelet Pain..

[62] Sorensen CJ, Norton BJ, Callaghan JP, Hwang C-T, Van Dillen LR (2015). Is lumbar lordosis related to low back pain development during prolonged standing?. Man Ther..

[63] Yu C, Zhan X, Liu C, Liao S, Xu J, Liang T (2020). Risk factors for recurrent L5-S1 disc herniation after percutaneous endoscopic transforaminal discectomy: a retrospective study. Med Sci Monit..

[64] Washburn SL (1960). Tools and human evolution. Sci Am..

[65] Lovejoy CO, Heiple KG, Burstein AH (1973). The gait of Australopithecus. Am J Phys Anthropol..

[66] Rosenberg KR (1992). The evolution of modern human childbirth. Am J Phys Anthropol..

[67] Ruff CB (1995). Biomechanics of the hip and birth in early Homo. Am J Phys Anthropol..

[68] Dunsworth HM, Trevathan WR, Rosenberg KR (2016). The obstetrical dilemma unraveled. Costly and cute: helpless infants and human evolution.

[69] Tague RG, Lovejoy CO (1986). The obstetric pelvis of A.L. 288-1 (Lucy). J Hum Evol..

[70] Laudicina NM, Rodriguez F, DeSilva JM (2019). Reconstructing birth in Australopithecus sediba. PLoS One..

[71] Berge C, Goularas D (2010). A new reconstruction of Sts 14 pelvis (Australopithecus africanus) from computed tomography and three-dimensional modeling techniques. J Hum Evol..

[72] Häusler M, Schmid P (1995). Comparison of the pelves of Sts 14 and AL288-1: implications for birth and sexual dimorphism in australopithecines. J Hum Evol..

[73] Been E, Gómez-Olivencia A, Kramer PA (2012). Lumbar lordosis of extinct hominins. Am J Phys Anthropol..

[74] Bailey JF, Sparrey CJ, Been E, Kramer PA (2016). Morphological and postural sexual dimorphism of the lumbar spine facilitates greater lordosis in females. J Anat..

[75] García-Martínez D, Martelli S, Torres-Tamayo N, Jiménez-Arenas JM, Martín AG, Campo M (2020). Sexual dimorphism in the vertebral wedging of the human lumbar vertebrae and its importance as a comparative framework for understanding the wedging pattern of Neanderthals. Quat Int.

[76] Kurki HK (2011). Pelvic dimorphism in relation to body size and body size dimorphism in humans. J Hum Evol..

[77] Mitteroecker P, Grunstra NDS, Stansfield E, Waltenberger L, Fischer B (2021). Did population differences in human pelvic form evolve by drift or selection?. Bulletins et mémoires de la Société d’anthropologie de Paris..

[78] Meredith HV (1970). Body weight at birth of viable human infants: a worldwide comparative treatise. Hum Biol..

[79] Mikolajczyk RT, Zhang J, Betran AP, Souza JP, Mori R, Gülmezoglu AM (2011). A global reference for fetal-weight and birthweight percentiles. Lancet..

[80] Villar J, Ismail LC, Victora CG, Ohuma EO, Bertino E, Altman DG (2014). International standards for newborn weight, length, and head circumference by gestational age and sex: the Newborn Cross-Sectional Study of the INTERGROWTH-21st Project. Lancet..

[81] Walker GJA, Gunasekera P (2011). Pelvic organ prolapse and incontinence in developing countries: review of prevalence and risk factors. Int Urogynecol J..

[82] Vergeldt TFM, Weemhoff M, IntHout J, Kluivers KB (2015). Risk factors for pelvic organ prolapse and its recurrence: a systematic review. Int Urogynecol J..

[83] Islam RM, Oldroyd J, Rana J, Romero L, Karim MN (2019). Prevalence of symptomatic pelvic floor disorders in community-dwelling women in low and middle-income countries: a systematic review and meta-analysis. Int Urogynecol J..

[84] Wells JCK, DeSilva JM, Stock JT (2012). The obstetric dilemma: an ancient game of Russian roulette, or a variable dilemma sensitive to ecology?. Am J Phys Anthropol..

[85] Zaffarini E, Mitteroecker P (2019). Secular changes in body height predict global rates of caesarean section. Proc Biol Sci..

[86] DelPrete H (2019). Similarities in pelvic dimorphisms across populations. Am J Hum Biol..

[87] Edgar HJH, Daneshvari Berry S, Moes E, Adolphi NL, Bridges P, Nolte KB (2020). New Mexico decedent image database.

[88] Mendez J (1960). Density and composition of mammalian muscle. Metabolism..

[89] Cobb WS, Burns JM, Kercher KW, Matthews BD, James Norton H, Todd HB (2005). Normal intraabdominal pressure in healthy adults. J Surg Res..

[90] Silva MET, Brandão S, Parente MPL, Mascarenhas T, Natal Jorge RM (2016). Establishing the biomechanical properties of the pelvic soft tissues through an inverse finite element analysis using magnetic resonance imaging. Proc Inst Mech Eng H..

[91] Silva MET, Brandão S, Parente MPL, Mascarenhas T, Natal Jorge RM (2017). Biomechanical properties of the pelvic floor muscles of continent and incontinent women using an inverse finite element analysis. Comput Methods Biomech Biomed Engin..

[92] Maas SA, Ellis BJ, Ateshian GA, Weiss JA (2012). FEBio: finite elements for biomechanics. J Biomech Eng..

[93] Oomens C, Brekelmans M, Baaijens F (2010). Biomechanics, concepts and computations.

[94] Bernstein IT (1997). The pelvic floor muscles: muscle thickness in healthy and urinary-incontinent women measured by perineal ultrasonography with reference to the effect of pelvic floor training. Estrogen receptor studies. Neurourol Urodyn..

[95] Tinelli A, Malvasi A, Rahimi S, Negro R, Vergara D, Martignago R (2010). Age-related pelvic floor modifications and prolapse risk factors in postmenopausal women. Menopause..

[96] Dehghan F, Haerian BS, Muniandy S, Yusof A, Dragoo JL, Salleh N (2014). The effect of relaxin on the musculoskeletal system. Scand J Med Sci Sports..

[97] Bhattarai A, Staat M (2018). Modelling of soft connective tissues to investigate female pelvic floor dysfunctions. Comput Math Methods Med..

[98] Bodner-Adler B, Alarab M, Ruiz-Zapata AM, Latthe P. Effectiveness of hormones in postmenopausal pelvic floor dysfunction-International Urogynecological Association research and development-committee opinion. Int Urogynecol J. 2019. 10.1007/s00192-019-04070-0. 10.1007/s00192-019-04070-0 PMC7363722 31392363

[99] Bø K, Nygaard IE (2020). Is physical activity good or bad for the female pelvic floor? A Narrative Review. Sports Med.

[100] Liapis I, Karachalios C, Bakas P, Panoulis K, Labrinoudaki I, Frangou-Plemenou M, et al. Expression and importance of relaxin in vaginal wall tissues from women with pelvic organ prolapse and with/without stress urinary incontinence. Clin Obstet Gynecol Reprod Med. 2020;6. 10.15761/cogrm.1000320.

[101] Ruiz-Zapata AM, Feola AJ, Heesakkers J, de Graaf P, Blaganje M, Sievert K-D (2018). Biomechanical properties of the pelvic floor and its relation to pelvic floor disorders. Eur Urol Suppl..

[102] Egorov V, Lucente V, VAN Raalte H, Murphy M, Ephrain S, Bhatia N (2019). Biomechanical mapping of the female pelvic floor: changes with age, parity and weight. Pelviperineology..

[103] El Sayed RF, Alt CD, Maccioni F, Meissnitzer M, Masselli G, Manganaro L (2017). Magnetic resonance imaging of pelvic floor dysfunction - joint recommendations of the ESUR and ESGAR Pelvic Floor Working Group. Eur Radiol..

[104] Fernández MM, Molina A, Valtorta Á (2015). Dynamic MRI of the pelvic floor: its usefulness at prolapse. Revista Argentina de Diagnóstico por Imágenes..

[105] Lockhart ME, Bates GW, Morgan DE, Beasley TM, Richter HE (2018). Dynamic 3T pelvic floor magnetic resonance imaging in women progressing from the nulligravid to the primiparous state. Int Urogynecol J..

[106] Arif-Tiwari H, Twiss CO, Lin FC, Funk JT, Vedantham S, Martin DR (2019). Improved detection of pelvic organ prolapse: comparative utility of defecography phase sequence to nondefecography valsalva maneuvers in dynamic pelvic floor magnetic resonance imaging. Curr Probl Diagn Radiol..

[107] Talasz H, Kremser C, Kofler M, et al. Phase-locked parallel movement of diaphragm and pelvic floor during breathing and coughing—a dynamic MRI investigation in healthy females. Int Urogynecol J. 2011;22:61–8. 10.1007/s00192-010-1240-z. 10.1007/s00192-010-1240-z 20809211

[108] Stansfield E, Grunstra ND, Fischer B, Pouca V, Mitteroecker P (2021). Dataset for “The evolution of pelvic canal shape and rotational birth in humans ”.

