# Supplementary material for: The evolution of pelvic canal shape and rotational birth in humans
Source: BMC Biol. 2021 Oct 11;19:224. doi: 10.1186/s12915-021-01150-w (PMC8507337; doi:10.1186/s12915-021-01150-w)
Supplement: Supplementary file 1 — Additional file 1: Tab. S1. List of models and their AP/ML ratios used for finite element analysis. Tab. S2. FE model validation. Fig. S1. Stress-strain relationships for the three geometries (i.e., flat, ellipsoid and anatomical) at the base dimensions. Fig. S2. Total displacement across all anatomical models. Fig. S3. Comparison of displacement maxima at the anterior and posterior compartments of the anatomical models and their total maxima. [file 12915_2021_1150_MOESM1_ESM.pdf]

Additional file 1

Creating model geometry

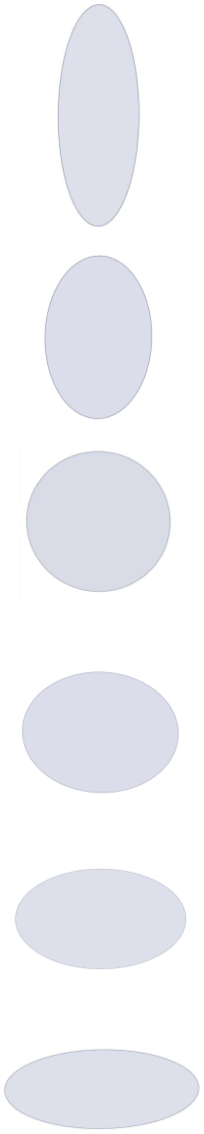

input values		flat model		ellipsoid model		anatomical model	
**SD**	**ML**	**AP**	**AP/ML**	**AP**	**AP/ML**	**AP**	**AP/ML**
**-4.50**	65.50	181.25	2.77	163.40	2.49	168.81	2.58
**-4.00**	70.00	169.60	2.42	156.60	2.24	160.60	2.29
**-3.50**	74.50	159.36	2.14	149.80	2.01	152.99	2.05
**-3.00**	79.00	150.28	1.90	143.70	1.82	145.91	1.85
**-2.50**	83.50	142.18	1.70	137.60	1.65	139.30	1.67
**-2.00**	88.00	134.91	1.53	132.00	1.50	133.13	1.51
**-1.50**	92.50	128.35	1.39	126.40	1.37	127.35	1.38
**-1.00**	97.00	122.39	1.26	121.70	1.25	121.92	1.26
**-0.50**	101.50	116.97	1.15	116.60	1.15	116.81	1.15
**0.00**	106.00	112.00	1.06	112.00	1.06	112.00	1.06
**+0.33**	108.97	108.97	1.00	108.97	1.00	108.97	1.00
**+0.67**	112.00	106.00	0.95	106.00	0.95	106.00	0.95
**+1.00**	115.00	103.23	0.90	103.10	0.90	103.16	0.90
**+1.50**	119.50	99.35	0.83	98.80	0.83	99.10	0.83
**+2.00**	124.00	95.74	0.77	95.00	0.77	95.24	0.77
**+2.50**	128.50	92.39	0.72	90.70	0.71	91.58	0.71
**+3.00**	133.00	89.26	0.67	87.20	0.66	88.10	0.66
**+3.50**	137.50	86.34	0.63	83.50	0.61	84.78	0.62
**+4.00**	142.00	83.61	0.59	80.30	0.57	81.63	0.57
**+4.50**	146.50	81.04	0.55	76.90	0.52	78.61	0.54
**+5.00**	151.00	78.62	0.52	73.80	0.49	75.73	0.50
**+6.00**	160.00	74.20	0.46	67.80	0.42	70.34	0.44
**+7.00**	169.00	70.25	0.42	62.10	0.37	65.40	0.39

Table S1. List of models and their AP/ML ratios used for finite element analysis. The column 'SD' indicates how many standard deviations were added to the mean of the ML (mediolateral) diameter (base model). The columns 'ML' and 'AP' indicate the mediolateral and anteroposterior diameters in mm; The ratios AP/ML are dimensionless.

Finite element model and its validation

We adopted an isotropic Mooney-Rivlin model (eq. 1) to represent pelvic floor tissues with the following parameters: c_1=26 kPa, c_2=14 kPa (*6*) and the bulk modulus, K = 1000 kPa to reflect the near-incompressibility of the material (*7*).

$$W = c_1(I_1 - 3) + c_2(I_2 - 3) + \frac{1}{2}K(\ln J)^2 \qquad \text{eq. (1)}$$

Here, c_1 and c_2 are the Mooney-Rivlin material coefficients, I_1 and I_2 are the invariants of the deviatoric part of the right Cauchy-Green deformation tensor, and J is the Jacobian of the deformation.

The Cauchy stress for the Mooney-Rivlin material under uniaxial loading depends on the stretch (λ) as

$$\sigma_{Mooney} = 2\left(\lambda^2 - \frac{1}{\lambda}\right)\left(c_1 + c_2\frac{1}{\lambda}\right) \qquad \text{eq. (2)}$$

where stretch (λ) is defined as the ratio of the deformed length over the original length.

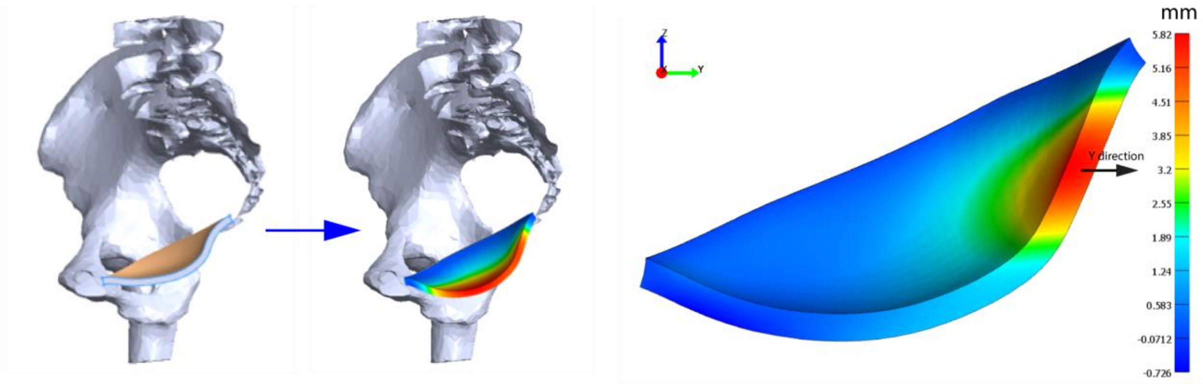

A **B**

	**Displacement (mm)**	
	**dMRI (Silva et al. 2017)**(*6*)	**FE base model**
**Y (horizontal)**	6.235±4.004	5.729
**Z (vertical)**	4.495±1.560	5.409

C

Table S2. FE model validation. (A) The overall change in shape change upon application of the pressure to the superior surface of the model. (B) Map of the displacement of the posterior compartment in the horizontal (Y) axis (in mm). We exported the average displacement of several elements at the posterior compartment. (C) Dynamic MRI (dMRI) and modelled Y and Z displacements of the posterior compartment for the base model.

Test of non-linear material effect

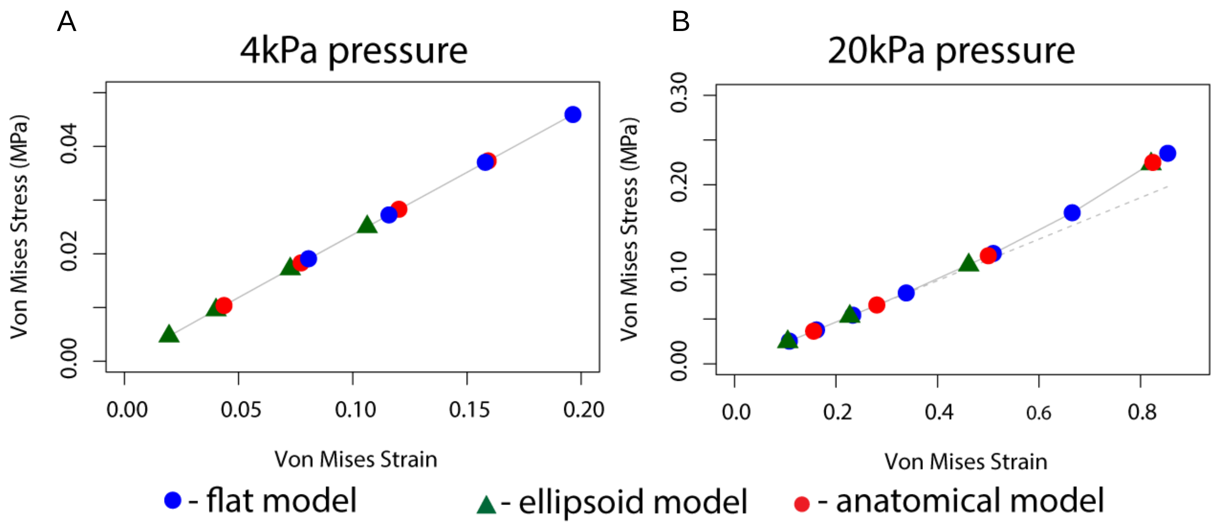

Figure S1. Stress-strain relationships for the three geometries (i.e., flat, ellipsoid and anatomical) at the base dimensions: (A) with applied pressure of 4 kPa; (B) with applied pressure of 20kPa. At 4kPa pressure, stress-strain relationships are linear. At 20kPa, the material becomes gradually stiffer. However, all models follow the same trajectory of the development of stiffness under the increase in strain. In (B), the dashed line shows an extrapolation of the linear relationship between stress and strain.

Maximum Total Displacement

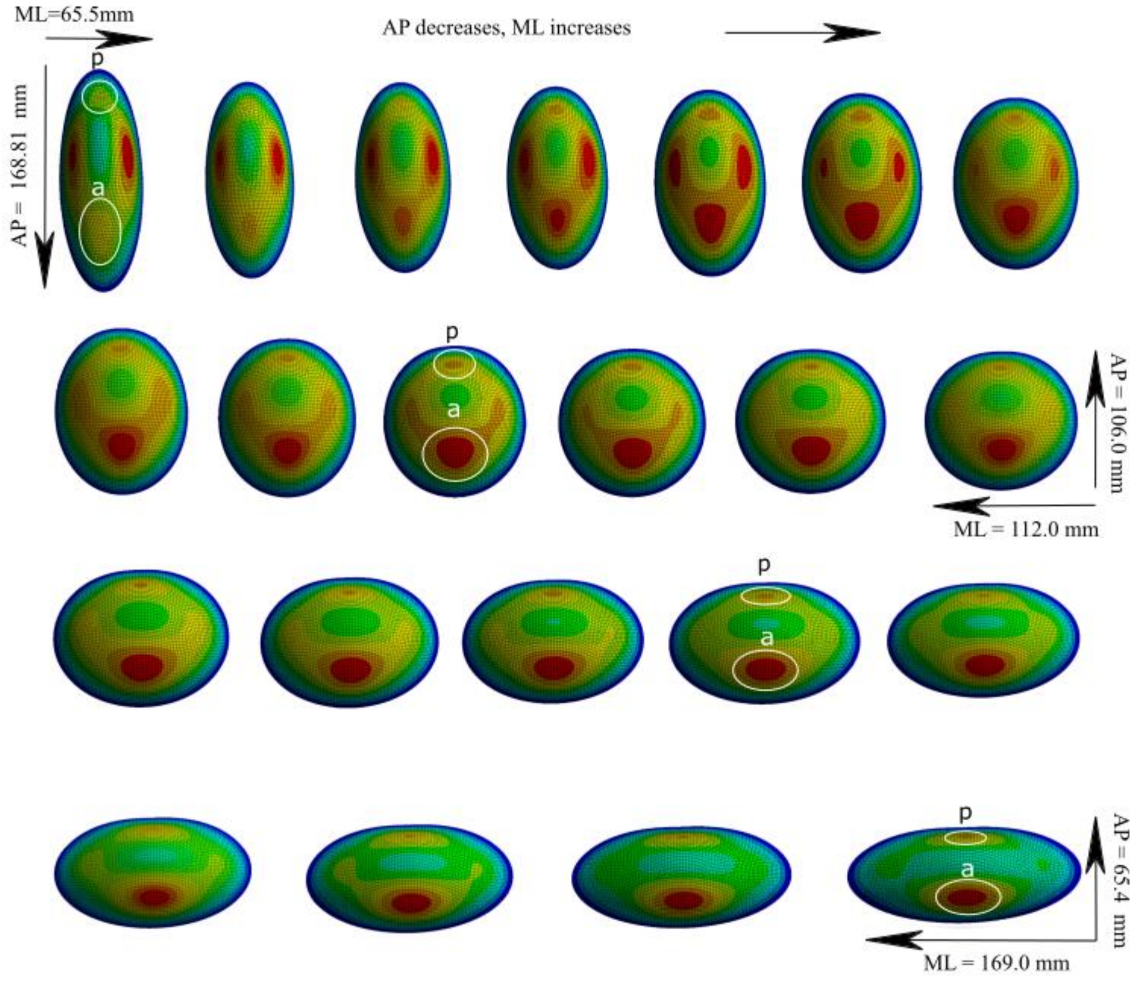

Figure S2. Total displacement across all anatomical models. White circles demarcate the position of the displacement centres for the anterior ("a") and posterior ("p") compartments. Unlike most shapes, the first four models with the largest AP/ML ratio display maxima of deformation outside of the two compartments.

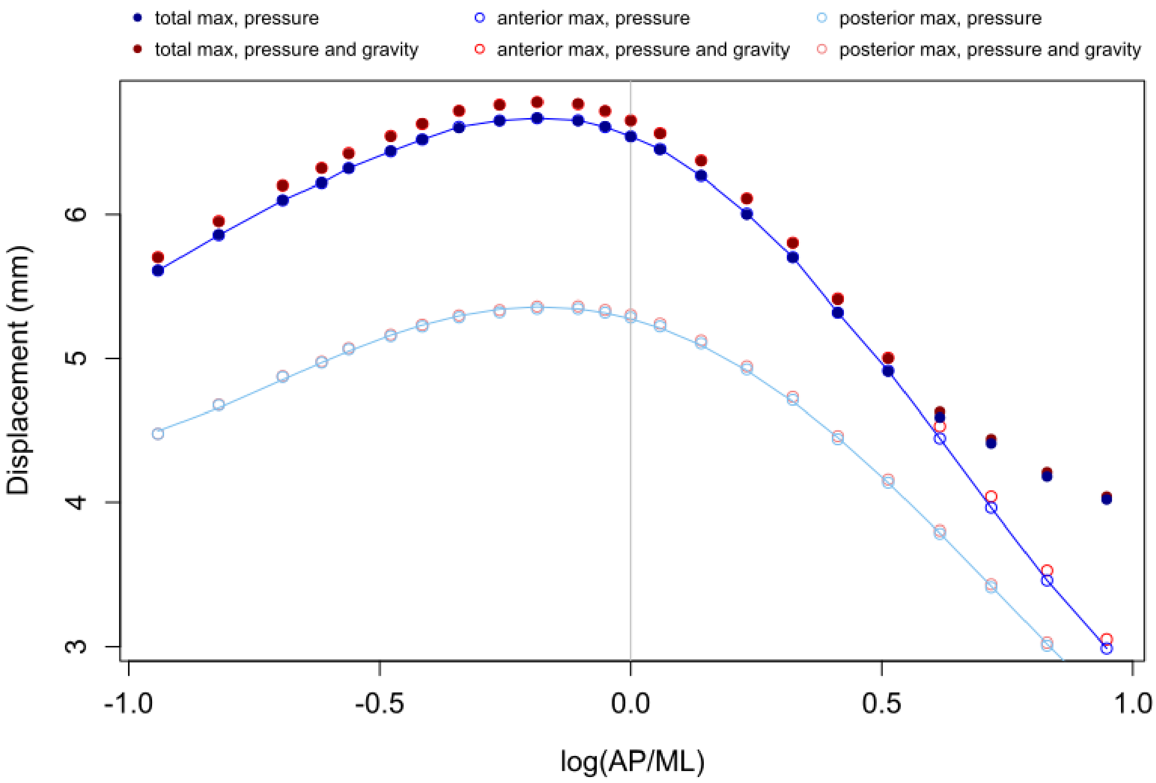

Figure S3. Comparison of displacement maxima at the anterior and posterior compartments of the anatomical models and their total maxima. The last four points on the right show results for models with the highest AP/ML ratio, the total maxima of which occur outside of the anterior and posterior compartment.
